# Assessment of Graphite, Graphene, and Hydrophilic-Treated Graphene Electrodes to Improve Power Generation and Wastewater Treatment in Microbial Fuel Cells

**DOI:** 10.3390/bioengineering10030378

**Published:** 2023-03-19

**Authors:** Fátima Borja-Maldonado, Miguel Ángel López Zavala

**Affiliations:** Tecnologico de Monterrey, School of Engineering and Sciences, Avenue Eugenio Garza Sada 2501, Monterrey 64849, Mexico

**Keywords:** anode, cathode, graphite, graphene, hydrophilic-treated graphene, microbial fuel cell

## Abstract

In this study, graphite, graphene, and hydrophilic-treated graphene electrodes were evaluated in a dual-chamber microbial fuel cell (DC-MFC). Free-oxygen conditions were promoted in anodic and cathodic chambers. Hydrochloric acid at 0.1 M and pH 1.1 was used as a catholyte, in addition to deionized water in the cathodic chamber. Domestic wastewater was used as a substrate, and a DuPontTM Nafion 117 membrane was used as a proton exchange membrane. The maximum power density of 32.07 mW·m${}^{-2}$ was obtained using hydrophilic-treated graphene electrodes and hydrochloric acid as catholyte. This power density was 1.4-fold and 32-fold greater than that of graphene (22.15 mW·m${}^{-2}$) and graphite (1.02 mW·m${}^{-2}$), respectively, under the same operational conditions. In addition, the maximum organic matter removal efficiencies of 69.8% and 75.5% were obtained using hydrophilic-treated graphene electrodes, for hydrochloric acid catholyte and deionized water, respectively. Therefore, the results suggest that the use of hydrophilic-treated graphene functioning as electrodes in DC-MFCs, and hydrochloric acid as a catholyte, favored power density when domestic wastewater is degraded. This opens up new possibilities for improving DC-MFC performance through the selection of suitable new electrode materials and catholytes.

## 1. Introduction

**Figure 1:**
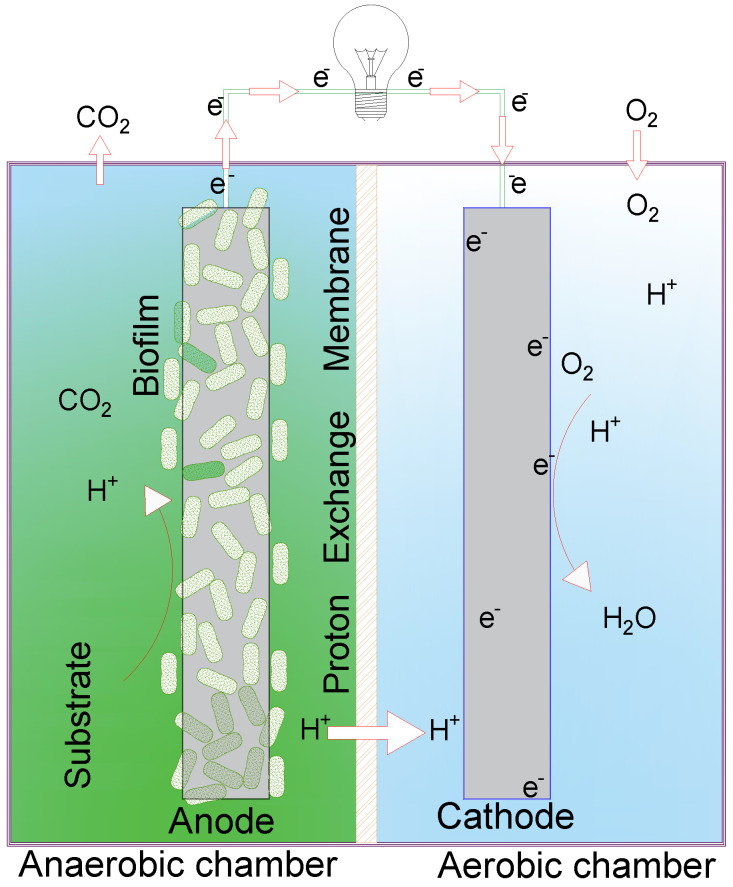
Dual−chamber microbial fuel cell.

Domestic wastewater contains a high level of organic compounds that store chemical energy, and with proper treatment, this energy can be harnessed [1]. Microbial fuel cells (MFCs) constitute a technology that can be applied to treat wastewater and simultaneously produce energy. The typical configuration is the DC-MFC, which consists of two chambers (anodic and cathodic) separated by a proton exchange membrane, as shown in Figure 1 [2]. An anode and a cathode which are connected by an external electric circuit are placed inside the chambers. A biofilm of exoelectrogenic microorganisms is formed on the surface of the anode. They obtain their energy from the oxidation of a substrate such as wastewater. This action releases protons, electrons, and carbon dioxide (CO${}_{2}$). Protons diffuse from the anodic chamber to the cathodic chamber through the membrane. The electrons are conducted from the anode (negative terminal) to the cathode (positive terminal) through an external electric circuit connected to an electrical resistance [2]. In the cathodic chamber, the electrons, protons, and oxygen (O${}_{2}$), present in conventional configurations, react to form water [3]. Energy production in MFCs is measured through the current or voltage generated, which are a function of the external resistance [2]. The efficiency of MFC systems is measured by their ability to degrade a substrate (such as wastewater) and generate energy.

Some strategies to maximize the benefits of DC-MFCs focus on user-controllable parameters such as electrode materials, substrate, catholyte, and device scale [4,5,6]. The materials used as electrodes have a direct effect on the performance of MFCs. Therefore, the proper selection of materials translates into a higher efficiency of the MFCs. In MFCs, the electrode can be made of carbon-based or metal-based materials. Even though metal-based materials have shown better properties than carbon-based materials, such as rapid oxidation-reduction reaction kinetics and high conductivity, they present some limitations such as high cost and low resistance to corrosion, which makes them difficult to consider on a large scale compared to laboratory devices. Therefore, carbon-based materials are most commonly used as electrodes in MFCs. For example, graphite is a widely used material, due to important advantages such as high biocompatibility, high availability, and low cost (compared to metal-based materials) [7].

Graphene is a material that has recently been used as anode and cathode in MFCs. Since its isolation in 2004, it has stood out for its unique properties. Graphene can be produced from graphite as the raw material, and then it is considered a low-cost material (compared to metal-based electrode materials). Its structure is a two-dimensional (2D) cohesion of carbon atoms, with a crystalline arrangement similar to a honeycomb. It can be found in nature in the form of powder or flakes [8]. This characteristic facilitates the transfer of electrons, which results in a high electrical conductivity at around 7200 S/m (compared to other materials, such as silicon), and high mechanical stability. Graphene has a large surface area compared to graphite (theoretical specific surface area of 2630 m${}^{2}$·g${}^{-1}$ and 10 m${}^{2}$·g${}^{-1}$, respectively) [9,10,11], thus favoring the interaction between the material and the electrolytes. Therefore, the selection, design, fabrication, and treatment of the electrode materials are crucial to improving its function as an anode or cathode.

The catholyte is another user-manipulable parameter, which influences the electron transfer, the participation of different final electron acceptors, and the reduction potential. Oxygen is the most widely used electron acceptor due to its unlimited availability and high standard redox potential. However, it is also a limiting factor in the performance of MFCs because it can have low oxygen reduction kinetics. To address this limitation, different catholytes with a higher redox potential that facilitate the availability of other electron acceptors have been used. Examples of these catholytes are ferricyanide, sodium hypochlorite (NaOCl), sodium bromate, and hydrochloric acid (HCl) [12,13,14,15]. López Zavala et al. [15] reported that using HCl as a catholyte in DC-MFC stimulated the hydrolysis of complex particles into soluble organic molecules, thus favoring the degradation of organic matter present in the substrate (wastewater). Recent studies of DC-MFCs reported the use of catholytes with the presence of metals that act as a final electron acceptor, while simultaneously the process is used to remove or recover metals. For instance, Kim et al. [16] evaluated a DC-MFC using wastewater containing chromium (Cr) VI as a catholyte that acted as a final electron acceptor, which was then reduced and precipitated, facilitating its removal. These strategies facilitate reduction kinetics by overcoming the activation energy and reducing cathodic activation loss.

To date, significant progress has been made in MFCs, however, there are still gaps to be addressed in order to improve energy efficiency and simultaneously the capacity to treat wastewater. Therefore, the objective of this work is to evaluate different carbon-based materials and the use of HCl acid as a catholyte to enhance the capacity of DC-MFCs to generate electricity and treat domestic wastewater and highlight the importance of identifying the optimal operating parameters, which directly influence MFC performance. For this purpose, three different carbon-based materials, graphite, graphene, and hydrophilic-treated graphene (HTG), were evaluated as electrodes in DC-MFCs. Additionally, HCl was evaluated as a catholyte, aiming to elucidate its influence on the catalytic properties of the cathodic chamber and to improve the degradation of organic matter in domestic wastewater. Furthermore, anoxic conditions were promoted in the anodic and cathodic chambers. Simultaneously, a control was run using the same electrode materials and under typical MFC operating conditions (deionized water in the cathodic chamber).

## 2. Materials and Methods

### 2.1. Characterization of Materials

The carbon-based materials used as electrodes were characterized by scanning electron microscopy (SEM) and Zeiss Evo ma24 energy dispersive X-ray spectroscopy (EDS) with a Bruker xflash6|10 detector, (Carl Zeiss, Microscopy Ltd., Cambridge, UK). The specific surface area and pore structure of the electrode materials were obtained with a Quantachrome Autosorb iQ tpx (Anton Paar, Monterrey, Mexico) by measuring the nitrogen (N${}_{2}$) adsorption/desorption isotherms, analyzed by the Brunauer–Emmett–Teller (BET) multipoint method, and the total pore volume by the density functional theory (DFT) technique. Raman spectra of the materials were measured at room temperature using a Renishaw inVia microspectrometer (Renishaw, Wotton-under-Edge, UK) with an excitation argon laser wavelength of 514 nm.

### 2.2. Experimental Device

**Figure 2:**
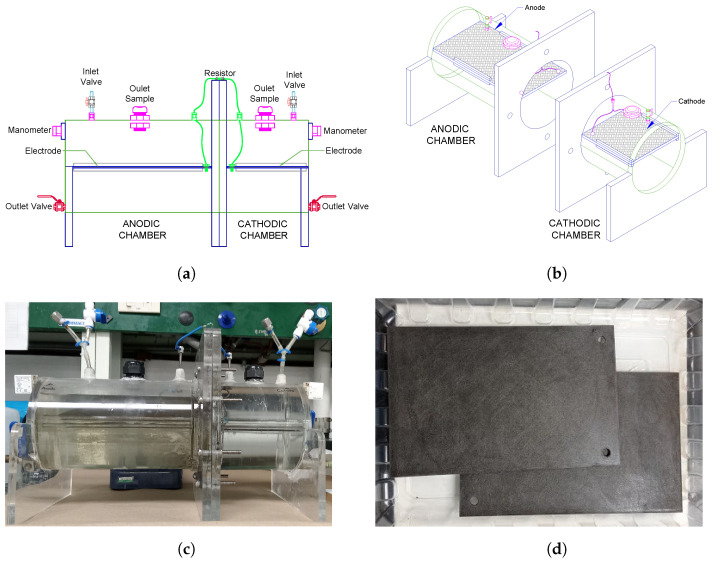
DC-MFC experimental device, (**a**) front view, (**b**) isometric view, (**c**) experimental system, and (**d**) graphite, graphene, and HTG electrodes.

The DC-MFC used in this study is shown in Figure 2a,b. The chambers were made with a 15.0 cm diameter and 6 mm thick acrylic tube (Figure 2c). The total volume of the chambers was 3.90 L for the anodic and 2.3 L for the cathodic chamber; each chamber has preparations for sampling the mixed liquor, electrolytes, and gases. The proton exchange membrane (PEM) used was a perfluorinated Nafion^®^ 117 (Sigma-Aldrich Quimica S de RL de CV, Toluca, Mexico). The membrane was activated before being used following the procedure described by Lavorante et al. [17] and Hasani-Sadrabadi et al. [18]. Graphite, graphene, and HTG (Figure 2d) were used as electrodes. The anode has dimensions of 21.0 × 13.4 cm and the cathode of 14.0 × 13.4 cm. The electrodes were connected through an external electrical circuit with AWG-16-gauge wire. The anode surface was sanded uniformly before each experiment, to facilitate the adherence of the anaerobic inoculum (biomass). The resistor used in the external electrical circuit had 100 Ω. The anodic chamber was filled with 2.40 L of domestic wastewater as a substrate and 1.0 L of conditioned inoculum. A magnetic stirrer and a digital stirring hotplate (Cimarec™Thermo Scientific, Monterrey, Mexico) were used to keep mixing conditions in the anodic chamber. The cathodic chamber was filled with 2.15 L of 0.1 M HCl solution (pH 1.11) as a catholyte. Nitrogen was bubbled to displace air and dissolve oxygen, followed by a vacuum (77.3 kPa), and then the chambers were closed to keep these conditions. In each experimental evaluation, a control MFC was set. The anodic chamber was operated under mixing conditions , the cathodic chamber was filled with deionized water and operated under atmospheric pressure conditions. A ProSense digital pressure sensor with a range of −100 kPa to 1 MPa was installed in each chamber to measure and monitor its pressure.

### 2.3. Inoculum Preparation

Mixed liquor and wastewater were taken from the aerobic bioreactor and the raw sewage collector, respectively, at the “Dulces Nombres” wastewater treatment plant of Monterrey city, Mexico. The mixed liquor was conditioned to obtain anaerobic biomass. First, the anode was placed in a 3.50 L container. Then, the container was filled with mixed liquor and fresh wastewater at a ratio of 1:2. Additionally, the pH of the wastewater was adjusted to a pH of 4.5 with HCl in order to promote the population of the acidogenic communities in the mixed liquor. After 24 h, the container was shaken vigorously; then the biomass was allowed to settle for 30 min. The container was opened to release the gases produced, and the supernatant was removed by decanting (avoiding biomass losses). Finally, the container was refilled with fresh wastewater. The above steps were repeated during 10 days at a temperature of 25 °C. After this time, the biomass was acclimated to anaerobic conditions with the biofilm attached to the anode surface, and it was ready to be used for testing in the DC-MFC [19]. The biomass amount was estimated by determining the volatile suspended solids according to the Solids-2540 Protocol of the Standard Methods for the Examination of Water and Wastewater [20].

### 2.4. Evaluation of MFC Performance

The evaluations of the carbon-based electrode materials were carried out in 6 scenarios, all in batch tests at 25 °C, using an external resistance of 100 Ω, and domestic wastewater as substrate. Scenario 1 evaluated graphite electrodes, scenario 2 evaluated graphene electrodes, and scenario 3 evaluated HTG electrodes. These scenarios were performed using HCl as catholyte and anoxic conditions in the anodic and cathodic chambers. Simultaneously, as a control, the same materials were evaluated but using deionized water in the cathodic chamber, designated as scenario 4 for graphite electrodes, scenario 5 for graphene electrodes, and scenario 6 for HTG electrodes. Every 24 h, a 20 mL supernatant sample was taken from the anodic chamber, using a syringe with a 15 cm 25-gauge needle. The organic load of the sample was then determined using the Reflux-titration closed Chemical Oxygen Demand (COD) 5520-C method and a Hach DRB200 Digital Reactor, described in Standard Methods for the Examination of Water and Wastewater [20].

The pH and conductivity of the samples were measured with a Thermo Scientific Orion Star A211 Benchtop pH Meter and a Thermo Scientific Orion 3 Star Conductivity Benchtop Meter (CTR Scientific, Monterrey, Mexico), respectively. Subsequently, with a hermetic micro-syringe fitted with a push-pull valve, a 5 mL sample of gases was taken from the chambers of the MFC. The gases generated (H${}_{2}$, CO${}_{2}$, and CH${}_{4}$) were analyzed with a MicroGC Agilent 490 gas chromatographed (Agilent Technologies, Mexico city, Mexico). The device was calibrated with a standard mixture of hydrogen (5%), oxygen (5%), nitrogen (5%), methane (5%), carbon dioxide (5%), and helium (75%) gases. The MFC potential was monitored with a Campbells Scientific A CR850 series datalogger. The current and power generated by the MFC were calculated with Equations (1) and (2), respectively, and the electrical energy generation with Equation (3).

(1) $$I=\frac{V}{R_{ext}}$$

(2) P=I×V

(3) E=P×t

where *I* is the current intensity in amperes (A), *V* is the potential in volts (V), $R_{ext}$ is the external resistance in ohms (Ω), *P* is the power in watts (W), *E* is the electrical energy in joules (J), and *t* is the time in seconds (s), COD. In addition, power density (mW·m${}^{-2}$ and mW·m${}^{-3}$) and Coulomb Efficiency (CE, %) were determined. CE is the ratio between the electrons transferred and the maximum value obtainable if all the oxidation of the substrate could produce a current. It was calculated as the ratio between the current flowing through the MFC and the theoretical current based on the total oxidation of the organic matter of the residual water in the anode chamber [2], with Equation (4).

(4) $$\mathrm{CE}=\frac{\int_{0}^{t}Idt}{FV_{I}\frac{(b_{0})\Delta COD}{M_{0}}}\;\times \;100$$

where *I* is the current intensity in amperes (A), *t* is the time in seconds (s), $M_{0}$ is the molecular weight of oxygen (32 g·mol${}^{-1}$), $V_{I}$ is the volume of liquor mixed in the anodic chamber in Liters (L), $b_{0}$ is the stoichiometric number of moles of electrons exchanged per mole of oxygen (4 mol e${}^{-}\cdot$mol${}^{-1}$), ΔCOD is the change in COD (g·L${}^{-1}$) at the time (*t*), *F* is the Faraday’s constant and represents the magnitude of electric charge per mole of electron (96,485 Coulomb·mol${}^{-1}$e${}^{-}$).

## 3. Results and Discussion

The evaluation of electrode materials was made based on the DC-MFC performance, taking into consideration energy production and COD removal.

### 3.1. Characterization of the Electrode Materials

**Figure 3:**
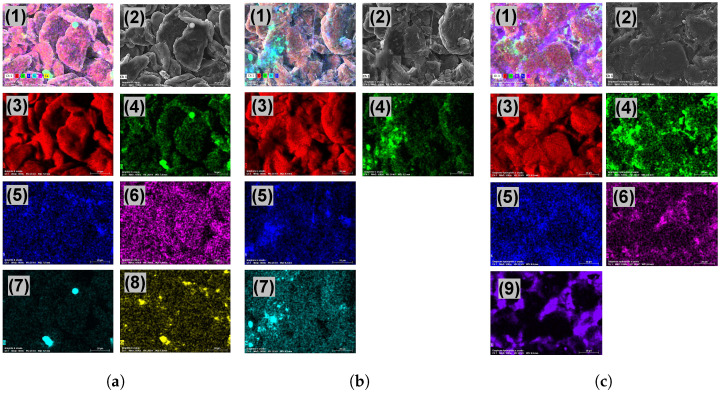
Energy dispersive X-ray spectroscopy (EDS) of electrode materials; (**a**) Graphite, (1) distribution of elements, (2) SEM Image, (3) carbon 89.81%, (4) oxygen 9.46%, (5) sulfur 0.13%, (6) phosphorus 0.12%, (7) silicon 0.22%, (8) calcium 0.26%; (**b**) Graphene, (1) distribution of elements, (2) SEM Image, (3) carbon 88.87%, (4) oxygen 10.40%, (5) sulfur 0.53%, (7) silicon 0.20%; (**c**) HTG, (1) distribution of elements, (2) SEM Image, (3) carbon 85.95%, (4) oxygen 7.39%, (5) sulfur 0.26%, (6) phosphorus 0.18%, (9) titanium 6.27%. The scale bar is 20 μm.

The electrode materials (graphite, graphene, and HTG) were analyzed to find out their properties. The configuration of the materials was in solid plates as shown in Figure 2d. Figure 3 shows the structure of the electrode materials analyzed by scanning electron microscope (SEM) and energy dispersive X-ray spectroscopy (EDS). Graphite mappings (Figure 3a) show the characteristic signal for carbon 89.81%, oxygen 9.46%, and in lower proportions, sulfur, phosphorus, silicon, and calcium, 0.12%, 0.22%, and 0.26%, respectively. Graphene mappings (Figure 3b) show a composition of carbon 88.87% and oxygen 10.40%, followed by sulfur, and silicon, 0.53%, and 0.2%, respectively. In both graphite and graphene, a distribution of carbon and oxygen typical of these materials was observed [21,22], and the presence of the other elements is attributed to depositions from wastewater to which the materials were exposed. Finally, Figure 3c shows the spectral map of the HTG with a homogeneous surface composition of carbon (85.55%) and oxygen (8.31%). In addition, visual evidence of the presence of titanium at 6.27% is given, which stands out as the main difference compared to the other two materials and could be attributed to possible doping applied to this material, as well as, in smaller proportions, the presence of sulfur and phosphorus in 0.26 and 0.18%, respectively, which are also depositions coming from the wastewater to which the material was exposed.

The specific surface area and pore structure of the electrode materials were obtained by measuring the adsorption/desorption isotherms of N${}_{2}$. The Brunauer–Emmett–Teller (BET) multipoint method was used to analyze specific surface areas, and the total pore volume was analyzed using the density functional theory (DFT) technique. The surface area of HTG, graphene, and graphite was 111.18, 39.66, and 20.15 (m${}^{2}$·g${}^{-1}$), respectively. These results show that the surface area of HTG was 2.80 times greater than that of graphene, and 5.50 times greater than graphite.

On the other hand, the pore volume of HTG, graphene, and graphite was 0.142, 0.030, and 0.032 (cm${}^{3}$·g${}^{-1}$), respectively. Some studies on graphene have reported changes in its surface area, pore size, or electrical conductivity when the material had been subjected to some type of modification. Zhao et al. [23] reported an increase in the surface area of graphene (from 2.39 to 279.00 m${}^{2}$·g${}^{-1}$) when this material was modified with titanium dioxide to obtain a new graphene nanostructure.

**Figure 4:**
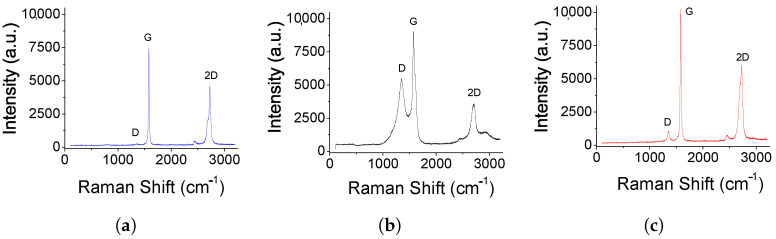
Comparison of Raman spectra of graphite (**a**), graphene (**b**), and HTG (**c**) electrode materials.

The electrode materials were also analyzed by Raman spectroscopy (Figure 4) with the same excitation laser to provide their probable number of graphene layers, structural defects, and doping of the material [24]. Figure 4a shows the structure of graphite, and, according to the literature, it matches the typical structural characteristics of graphite. The Raman spectra include two bands, the G-1581 cm${}^{-1}$ band, assigned to carbon atoms, and the D-1350 cm${}^{-1}$ band, assigned to structural disorder. Therefore, the ordered or high crystallinity structure confirms that the material evaluated is graphite [25,26]. Figure 4b shows the spectra of graphene. It is observed that the D-band associated with disorder increases in width and intensity, and the G-band increases and shifts slightly at high frequencies. This increase in the intensity of the D-band indicates the introduction of defects in its structure. Figure 4c shows the HTG spectra, and it is evident that the material presents a difference in its structure compared to graphene. The Raman of HTG shows a less intense D-band than that of graphene, which means that graphene has more disorder in its structure. Likewise, more structural disorder is observed in graphene compared to graphite, which is a typical characteristic of these materials. Further, HTG shows a structure with higher crystallinity than graphene, which can be attributed to the titanium depositions on the surface of HTG (Figure 3c). The 2D-band is mainly used to identify if graphene has a single-layer, bi-layer, or multi-layer of carbon atoms. HTG shows higher and slightly broader intensity in the 2D-band than graphene, indicating that graphene contains sheets with a single or two mono-layers of carbon atoms, whereas HTG contains few layers [27,28,29,30].

### 3.2. Wastewater Biodegradation

**Figure 5:**
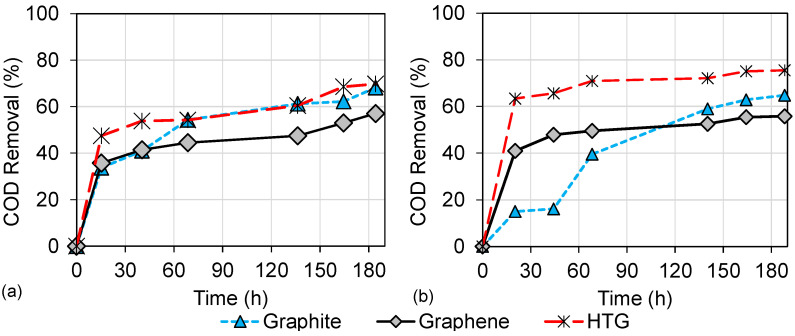
COD removal in the DC-MFC for different electrode materials. (**a**) HCl as a catholyte; (**b**) deionized water in the cathodic chamber.

The ability of the DC-MFCs systems to degrade organic matter in wastewater using graphite, graphene, and HTG is shown in Figure 5. Figure 5a shows the maximum COD removal efficiencies of 68.19%, 57.09%, and 69.78% for graphite, graphene, and HTG, respectively, when the HCl was used as a catholyte; meanwhile, Figure 5b shows the maximum COD removal efficiencies of 64.81%, 55.85%, and 75.51% for graphite, graphene, and HTG, respectively, when deionized water as used in the cathodic chamber. The highest COD removal (75.51%) was obtained when HTG electrodes and deionized water were used, followed by scenario 3 where HTG and HCl as a catholyte were used. These results can be attributed to the specific properties obtained in HTG, such as titanium depositions, higher surface area, and pore volume, compared to the results obtained with graphite and graphene, shown in the previous Section 3.1, which promote a higher availability of the active site for microorganisms interaction in HTG material. As has been reported, graphene electrodes can favor anode–microorganisms interaction and instantaneously promote biomass growth and enhance direct electron transfer [9,31]. In addition, it has been reported that titanium or titanium dioxide can promote the surface roughness, hydrophilicity, and conductive properties of the anode, as well as promoting the interaction between and performance level of the electrochemically active biofilm and the anode surface [10,32,33].

**Figure 6:**
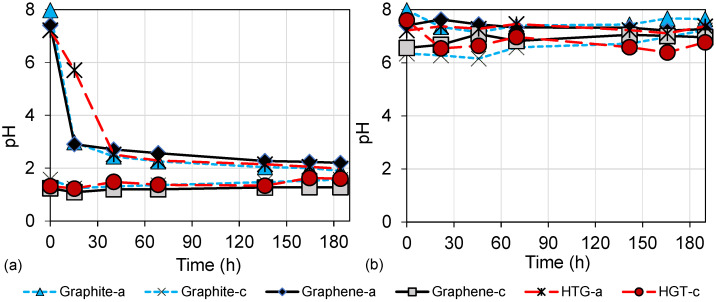
Evolution of the pH over time. (**a**) pH of the anodic and cathodic chamber using graphite, graphene, and HTG electrodes under HCl as a catholyte; (**b**) pH of the anodic and cathodic chamber using graphite, graphene, and HTG electrodes under deionized water in the cathodic chamber.

Another parameter that influences the COD removal and the overall DC-MFC performance is the pH in both the anodic and the cathodic chambers. Figure 6 shows the evolution of pH over time in the evaluations of the electrode materials. In scenarios 1, 2, and 3 operated with HCl as the catholyte (Figure 6a), it was observed that in the first 48 h, the pH values of the anodic chamber decreased from initial values between 7 and 8 to pH values close to 3. The decrease in pH occurred due to the diffusion of HCl from the cathodic chamber to the anodic chamber through the semipermeable membrane Nafion 117. The pH gradually changes until it converges in both chambers [34]. This pH behavior occurs when the pH values are different in the anode and cathode chambers. On the other hand, in scenarios 4, 5, and 6 operated with deionized water (Figure 6b), the pH was maintained with minimal changes in both chambers. These results can be linked to the performance of organic matter degradation in each scenario.

In the first instance, scenarios 1 and 2 using graphite and graphene electrodes, and operated with HCl as a catholyte show higher COD removal (68.19% and 57.09%, respectively) than scenarios 4 and 5 using deionized water. This performance can be attributed to the acidification of the anodic chamber, which can favor the hydrolysis of organic matter whereby organic polymers can be biologically decomposed into smaller molecules (such as carbohydrates, lipids, and proteins) that are able to cross the cell membrane. These smaller molecules can then be transformed into sugars, long-chain fatty acids, and amino acids [35]. Furthermore, this low pH can decrease or even inhibit the growth of other microorganisms, such as methanogens, and promote the development of microorganisms resistant to the low pH. These latter microorganisms include acidophilic microorganisms, e.g., of the acidogenic and acetogenic type, that prefer environments with a pH below 5.5 [36,37,38]. This improvement in COD removal capacity in DC-MFC using HCl as a catholyte has also been observed in the previous study by López Zavala et al. [15]. In this study, scenarios 1 and 2 reported similar behavior that suggests an advantage of using HCl as a catholyte.

In contrast, scenarios 3 and 6 using HTG showed different behavior, as the highest (of all scenarios) COD removal of 75.51% was obtained in scenario 6 operated with deionized water in the cathodic chamber, followed by scenario 3 operated with HCl as a catholyte. In this case, the combination of HTG electrodes under acid conditions can cause a slight increase in overpotentials due to activation losses in DC-MFC. These activation losses in acidophilic microorganism communities are higher because of the amount of energy required to maintain their internal cytoplasmic pH [39]. Likewise, the energy required to initiate oxidation and reduction reactions, and to transfer electrons to the anode surface, is greater in acidophilic microorganisms than in mixed microorganism communities developed under neutral conditions such as those promoted in scenario 6.

Mass transfer losses are another factor that can influence the COD removal in scenario 3 (HTG electrodes and acid conditions) since the decrease in pH can generate an accumulation of protons which can have an adverse effect on bacterial kinetics [40], as well as a possible antibacterial effect of HTG through the physical damage to the microorganisms produced by possible sharp edges of the material and therefore a decrease in the digestive activity of the biofilm [41].

**Figure 7:**
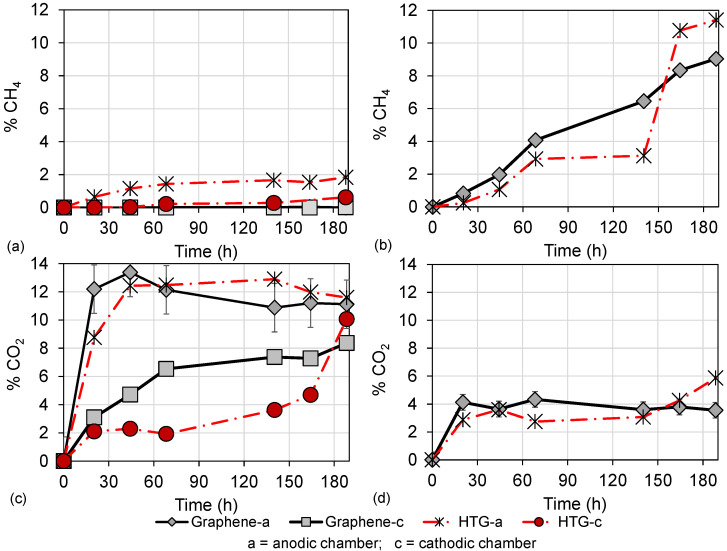
Production of gases in DC-MFC. (**a**) Methane (CH${}_{4}$) gas of the anodic and cathodic chamber using graphene and HTG electrodes under HCl as a catholyte; (**b**) methane (CH${}_{4}$) gas of the anodic and cathodic chamber using graphene and HTG electrodes under deionized water in the cathodic chamber; (**c**) methane (CO${}_{2}$) gas of the anodic and cathodic chamber using graphene and HTG electrodes under HCl as a catholyte; (**d**) methane (CO${}_{2}$) gas of the anodic and cathodic chamber using graphene and HTG electrodes under deionized water in the cathodic chamber.

Regarding gas production, Figure 7 shows gas detection over time in the graphene and HTG evaluations. Figure 7a shows the methane (CH${}_{4}$) production of scenarios 2 and 3 evaluated with HCl as catholyte, which did not exceed 2%. This low detection of CH${}_{4}$ gas is attributed to the growth inhibition of methanogenic consortia, since these microorganisms are neutrophilic and grow better at pH values between 5.5 and 8, and environments with pH outside this range are unfavorable for their development [42]. In the scenarios where HCl was used as a catholyte, a diffusion of HCl through the membrane was observed. This results in a decrease in the pH of the anodic chamber, to values around pH 2 (Figure 6), which generated an acidic environment adverse for methanogenic microorganisms.

Furthermore, in Figure 7c the detection of carbon dioxide (CO${}_{2}$) over time is shown. The maximum CO${}_{2}$ detection, close to 12%, is obtained in scenario 3 (using HTG electrodes and HCl). The behavior of CO${}_{2}$ gas in both anodic and cathodic chambers is upward with a tendency to converge, which is attributed to gas diffusion through the membrane. These results also suggest that COD degradation happens mainly through acidophilic microorganisms (from environments with pH < 5.5). This is confirmed because simple compounds, including CO${}_{2}$, are produced in the acidophilic phase of anaerobic degradation. Therefore, a higher detection of CO${}_{2}$ and lower or no detection of CH${}_{4}$ is observed in scenarios 2 and 3, which operated with HCl as catholyte.

The opposite behavior, higher CH${}_{4}$ detection and lower CO${}_{2}$ detection was observed in scenarios 5 and 6, which operated with deionized water in the cathodic chamber. Figure 7b shows that the maximum CH${}_{4}$ detection was close to 12% using HTG electrodes. Therefore, it was observed that since there was no significant pH shift in DC-MFC (scenarios 5 and 6 with pH between 6.5 and 8.0, Figure 6), the growth of the methanogenic population and consequently the CH${}_{4}$ gas was favored. In this methanogenic stage of degradation, it is possible to obtain the growth of these microorganisms by different routes, such as the hydrogenotrophic phase [37]. In this route, the microorganisms use CO${}_{2}$ and hydrogen as a substrate and degrade it to finally produce CH${}_{4}$. This may explain the results observed in scenarios 5 and 6 (Figure 7b,d), where a higher detection of CH${}_{4}$ was observed compared to a low detection of CO${}_{2}$.

Hence, these results show that the highest percentage of COD removal was obtained in scenario 6 (with HTG electrodes and deionized water in the cathodic chamber), indicating that degradation was mainly carried out by methanogenic consortia. In second place, with a slightly lower COD removal percentage, scenario 3 (with HTG electrodes and HCl as catholyte) stands out, indicating that degradation was mainly carried out by acidogenic consortia. Consequently, although HCl as a catholyte or deionized water in the cathodic chamber was used in the DC-MFC evaluations, HTG electrodes are the ones that most favor COD degradation compared to the other electrode materials, graphene, and graphite. In order to identify the operating conditions that most favor the overall performance of the DC-MFC, the electrical results of each scenario were analyzed and linked with those mentioned above.

### 3.3. Energy Production in DC-MFCs

**Figure 8:**
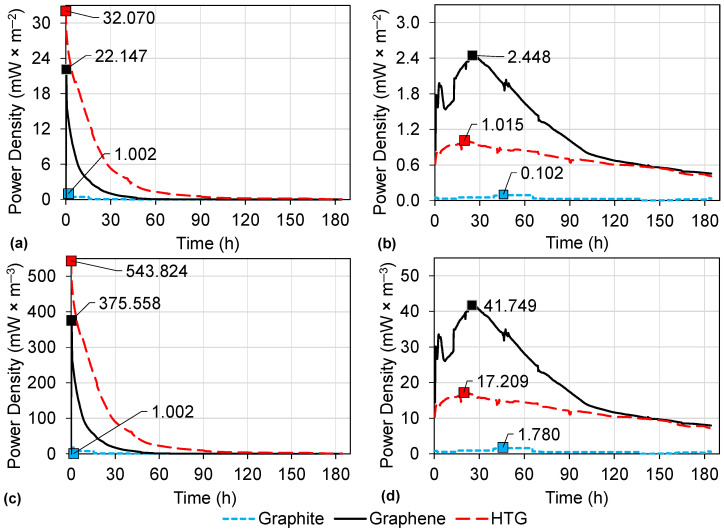
Power density of the DC-MFC using different conditions. (**a**) Power density per area using HCl catholyte; (**b**) power density per area using deionized water; (**c**) power density per volume using HCl catholyte; (**d**) power density per volume using deionized water.

In each scenario described in Section 2.4, energy production was monitored and analyzed according to the corresponding Equations (1)–(4). The comparison of the power density produced by graphite, graphene, and HTG electrodes using an external resistance of 100 Ω in HCl and deionized water is shown in Figure 8. The power densities were normalized to the surface area of the electrode and to the total volume of the anodic chamber. The maximum power densities obtained from the evaluations of graphite, graphene, and HTG electrodes using HCl as a catholyte were 1.002, 22.147, and 32.07 mW·m${}^{-2}$, respectively (see Figure 8a); and 1.002, 375.56, and 543.82 mW·m${}^{-3}$, respectively (Figure 8c). On the other hand, the maximum power densities from graphite, graphene, and HTG electrodes using deionized water in the cathodic chamber were 0.102, 2.448 and 0.973 mW·m${}^{-2}$, respectively (Figure 8b); and 1.78, 41.75, and 17.21 mW·m${}^{-3}$, respectively (Figure 8d).

According to these results, scenario 3 with HTG as anode and cathode and HCl as catholyte (Table 1) stood out, with a maximum power density of 32.07 mW·m${}^{-2}$. This power density is 1.4-fold higher than the second-highest power density obtained with graphene electrodes and HCl catholyte (22.147 mW·m${}^{-2}$, scenario 2). It was also observed that these results (32.07, and 22.147 mW·m${}^{-2}$) were considerably higher, on the order of 11-fold, than the power densities obtained in the other evaluations using graphite, graphene, and HTG from scenarios 1, 4, 5, and 6 shown in Table 1, which did not exceed 3 mW·m${}^{-2}$. This higher power density in scenario 3 (Table 1) can be attributed to the reduction in ohmic losses, that is, a reduction in resistance to electron flow through the anolyte and catholyte, electrodes, and electrical connections [2,43], while the other operating conditions in scenarios 1, 4, 5, and 6 are more susceptible to the adverse effects of losses.

**Figure 9:**
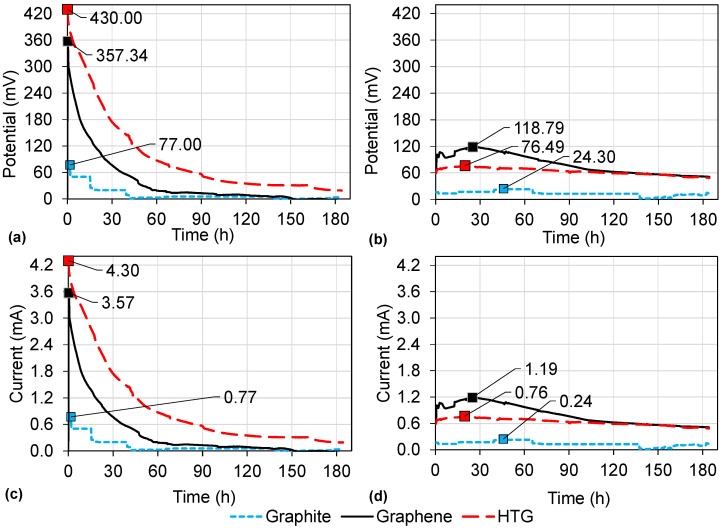
Electricity generation with graphite, graphene, and HTG electrode materials: (**a**) potential production using HCl catholyte; (**b**) potential production using deionized water in the cathodic chamber; (**c**) intensity production using HCl catholyte; (**d**) intensity production using deionized water in the cathodic chamber.

In Figure 9a,b, the potential of all the electrode evaluations is shown. The highest potential (430 mV) was obtained in the assessment of HTG using a HCl catholyte. Further, the current is shown in Figure 9c,d exhibiting a maximum of 4.30 mA in the same scenario of HTG using a HCl catholyte.

**Figure 10:**
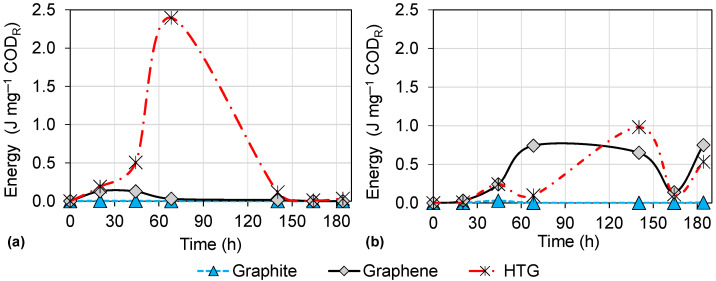
Correlation between energy production and organic matter removed with graphite, graphene, and HTG electrode materials: (**a**) using HCl catholyte; (**b**) using deionized water in the cathodic chamber. COD${}_{R}$ = chemical oxygen demand reduced.

Additionally, the energy production (Figure 10) showed the highest value of 2.40 J·mg${}^{-1}$ COD reduced. Therefore, it is highlighted that HTG electrodes with HCl as catholyte are the conditions that most favor electrical production, followed by graphene electrodes, also with a HCl catholyte. This may be associated whit the fact that HCl dissociates almost completely in aqueous solutions, which makes it an excellent conductor of electricity and a good electrolyte [44]. Moreover, the low pH (Figure 6) in the anodic chamber as a result of the diffusion of the catholyte through the membrane could contribute to generation of electricity, since pH in MFCs is a parameter that can affect the energy production and the growth of electrogenic microorganisms (microorganisms that oxidize a substrate and release electrons that can be transferred to an external medium [7]) [45]. Jang et al. [46] also reported an improvement in the current when they used HCl in the cathodic chamber compared to the use of sodium chloride (NaCl), and they attributed this to the increase in the protons availability to the cathode.

In addition, the HTG electrodes exhibited the maximum power output in DC-MFC, compared to the untreated graphene, and graphite electrodes. This can be attributed to the fact that the Ti depositions on the HTG participate as optimizers of graphene features including electrical conductivity and the surface area of the material, as confirmed in the previous Section 3.1. These properties could improve the effective biofilm attachment and power density. Similar results were also reported by Zhao et al. [23]. They evaluated the use of a titanium dioxide-graphene anode used in MFCs and obtained an improvement in bacterial adhesion and power density.

Table 1 summarizes the maximum values of potential, current, power density, and COD obtained in each scenario. In the overall analysis in Table 1, the maximum power density obtained (32.07 mW·m${}^{-2}$) in scenario 3 with HTG achieved a COD removal of 69.78%. On the other hand, when deionized water with HTG electrodes was used (scenario 6), a lower power density (0.973 mW·m${}^{-2}$), but slightly higher degradation (75.51%) was obtained compared to scenario 3. These results in scenario 6 suggest that the degradation of organic matter was carried out by other non-electrogenic microorganisms. On the contrary, in scenario 3 it is evident how the use of HCl as a catholyte significantly favors energy production. However, as mentioned in the previous Section 3.2 “Wastewater degradation”, it can also generate a slight antibacterial effect which in turn produces a decrease in the activity of the biofilm.

Few studies have reported the use of a low-pH environment in MFCs: Jannelli et al. [47] evaluated MFCs of 50 mL operated at pH 3.0 ± 0.5 using NaCl as catholyte and reported a power density around 20 to 55 mW·m${}^{-2}$ and COD removal of only 45% at 28 operation days. However, they indicated that the use of this acidic condition limited the MFCs’ performance. Ni et al. [38] evaluated the feasibility of MFCs to degrade wastewater from an industrial sulfide mineral flotation and produce energy through the use of acidophilic microorganisms (under pH 2 using sulfuric acid). They reported that is possible to generate a maximum voltage of 105 ± 42 mV and a power density of 4.8 mW·m${}^{-2}$. In low-pH environments, there is a greater availability of protons and thus the current can be higher compared to the energy obtained in MFCs at neutral pH [48,49,50]. This makes it evident that the biofilms of MFCs can adapt to low pH and produce electrical energy, making it possible to use them for the bioremediation of acid wastewater such as acidic mining streams.

Hence, the use of HTG or graphene as electrodes, both operated under acidic conditions (HCl as catholyte), stand out as the scenarios that best favor energy production in DC-MFCs. Regarding the efficiencies obtained in scenarios 2 and 3 to degrade the organic matter in the wastewater, it is suggested to analyze new scenarios with a lower concentration of HCl as the catholyte. The aim is to take advantage of the benefits to produce electrical energy, and at the same time decrease the possible adverse effects on the electrogenic microorganisms and increase the capacity to oxidize the substrate.

## 4. Conclusions

This study evaluated three electrode materials (graphite, graphene, and hydrophilically treated graphene (HTG)) in DC-MFC in the capacity of treating 3.40 L of domestic wastewater and using HCl (0.1 M HCl, pH 1.1) as a catholyte, and deionized water in the cathodic chamber. A comparison of electrode materials, taking into account both their ability to produce energy and to treat wastewater, ranked them from highest to lowest performance in the following order: HTG, graphene, and graphite using HCl as catholyte. According to the characterization of the HTG material, a larger surface area, large pore volume, and higher electrical conductivity were observed, which can be attributed to the Ti deposition found on its surface. These properties in the HTG promoted the availability of active sites and, in turn, the availability of improved adhesion of the electrogenic microorganisms, resulting in the better overall performance of the DC-MFCs. In the scenario with HTG electrodes, HCl as catholyte, and an external resistance of 100, the maximum power density of 32.07 mW·m${}^{-2}$ was obtained, which was 1.4-fold and 32-fold times higher than that of graphene and graphite, respectively. Regarding wastewater treatment, 69.8% and 75.5% were obtained using HTG electrodes, for HCl catholyte and deionized water in the cathodic chamber, respectively. Hence, although the highest COD removal was not obtained in the scenario where the highest power density was obtained, the best overall balance of the DC-MFC considering both parameters was achieved in the scenario using HTG electrodes and HCl as catholyte. Further studies of DC-MFC with HTG electrodes are recommended to evaluate different scenarios in order to identify the optimal operating conditions to increase the ability to remove COD.

## Figures and Tables

**Table 1:** Energy production in the DC-MFC for graphite, graphene, and HTG, under different catholytes.

Sc	Material		Catholyte	Voltage	Current	Density Power	COD
	Anode	Cathode		mV	(mA)	mW·m${}^{-2}$	Removal (%)
1	Graphite	Graphite	HCl	77.00	0.770	1.002	68.19
2	Graphene	Graphene	HCl	357.34	3.573	22.147	57.09
3	HTG	HTG	HCl	430.00	4.300	32.070	69.78
4	Graphite	Graphite	DW	24.30	0.243	0.102	64.81
5	Graphene	Graphene	DW	118.79	1.188	2.448	55.85
6	HTG	HTG	DW	74.90	0.749	0.973	75.51

Sc = scenario; HTG = hydrophilic-treated graphene; DW = deionized water.

## Data Availability

Data is unavailable due to privacy or ethical restrictions.

## Abbreviations

The following abbreviations are used in this manuscript:

HTG	Hydrophilic-treated graphene
DC-MFC	Dual chamber microbial fuel cell
HCl	Hydrochloric acid
COD	Chemical Oxygen Demand
CE	Coulombic efficiency
Ti	Titanium

## References

[1] Yang Y., Liu T., Zhu X., Zhang F., Ye D., Liao Q., Li Y. (2016). Boosting Power Density of Microbial Fuel Cells with 3D Nitrogen-Doped Graphene Aerogel Electrode. Adv. Sci..

[2] Logan B.E., Hamelers B., Rozendal R., Schröder U., Keller J., Freguia S., Aelterman P., Verstraete W., Rabaey K. (2006). Microbial Fuel Cells: Methodology and Technology. Environ. Sci. Technol..

[3] Min B., Logan B.E. (2004). Continuous Electricity Generation from Domestic Wastewater and Organic Substrates in a Flat Plate Microbial Fuel Cell. Environ. Sci. Technol..

[4] Menicucci J., Beyenal H., Marsili E., Veluchamy, Demir G., Lewandowski Z. (2006). Procedure for Determining Maximum Sustainable Power Generated by Microbial Fuel Cells. Environ. Sci. Technol..

[5] González del Campo A., Cañizares P., Lobato J., Rodrigo M., Fernandez Morales F.J. (2016). Effects of External Resistance on Microbial Fuel Cell’s Performance.

[6] Song T.S., Yan Z.S., Zhao Z.W., Jiang H.L. (2010). Removal of organic matter in freshwater sediment by microbial fuel cells at various external resistances. J. Chem. Technol. Biotechnol..

[7] Borja-Maldonado F., Zavala M.Á.L. (2022). Contribution of configurations, electrode and membrane materials, electron transfer mechanisms, and cost of components on the current and future development of microbial fuel cells. Heliyon.

[8] Pavoski G., Maraschin T., Fim F.d.C., Balzaretti N.M., Galland G.B., Moura C.S., de Souza Basso N.R. (2016). Few Layer Reduced Graphene Oxide: Evaluation of the Best Experimental Conditions for Easy Production. Mater. Res..

[9] ElMekawy A., Hegab H.M., Losic D., Saint C.P., Pant D. (2017). Applications of graphene in microbial fuel cells: The gap between promise and reality. Renew. Sustain. Energy Rev..

[10] Tang J., Yuan Y., Liu T., Zhou S. (2015). High-capacity carbon-coated titanium dioxide core-shell nanoparticles modified three dimensional anodes for improved energy output in microbial fuel cells. J. Power Sources.

[11] Huang X., Zeng Z., Fan Z., Liu J., Zhang H. (2012). Graphene-Based Electrodes. Adv. Mater..

[12] Lawson K., Rossi R., Regan J.M., Logan B.E. (2020). Impact of cathodic electron acceptor on microbial fuel cell internal resistance. Bioresour. Technol..

[13] Jadhav D., Ghadge A., Mondal D., Ghangrekar M. (2014). Comparison of oxygen and hypochlorite as cathodic electron acceptor in microbial fuel cells. Bioresour. Technol..

[14] Dai H., Yang H., Liu X., Zhao Y., Liang Z. (2016). Performance of sodium bromate as cathodic electron acceptor in microbial fuel cell. Bioresour. Technol..

[15] López Zavala M., Torres Delenne P., González Peña O. (2018). Improvement of Wastewater Treatment Performance and Power Generation in Microbial Fuel Cells by Enhancing Hydrolysis and Acidogenesis, and by Reducing Internal Losses. Energies.

[16] Kim C., Lee C.R., Song Y.E., Heo J., Choi S.M., Lim D.H., Cho J., Park C., Jang M., Kim J.R. (2017). Hexavalent chromium as a cathodic electron acceptor in a bipolar membrane microbial fuel cell with the simultaneous treatment of electroplating wastewater. Chem. Eng. J..

[17] Napoli L., Lavorante M., Franco J., Sanguinetti A., Fasoli H. (2013). Effects on Nafion^®^ 117 Membrane using Different Strong Acids in Various Concentrations. J. New Mater. Electrochem. Syst..

[18] Hasani-Sadrabadi M.M., Dashtimoghadam E., Majedi F.S., Kabiri K., Solati-Hashjin M., Moaddel H. (2010). Novel nanocomposite proton exchange membranes based on Nafion^®^ and AMPS-modified montmorillonite for fuel cell applications. J. Membr. Sci..

[19] Rodrigo M., Cañizares P., Lobato J., Paz R., Sáez C., Linares J. (2007). Production of electricity from the treatment of urban waste water using a microbial fuel cell. J. Power Sources.

[20] Eaton A., Clesceri L., Franson M., Rice E., Greenberg A., Association, American Public Health, Association, American Water Works, Federation, Water Environment (2005). Standard Methods for the Examination of Water & Wastewater.

[21] Siburian R., Sihotang H., Lumban Raja S., Supeno M., Simanjuntak C. (2018). New Route to Synthesize of Graphene Nano Sheets. Orient. J. Chem..

[22] Kumara G.R.A., Pitawala H.M.G.T.A., Karunarathne B., Mantilaka M.M.M.G.P.G., Rajapakse R.M.G., Huang H.H., De Silva K.K.H., Yoshimura M. (2021). Development of a chemical-free floatation technology for the purification of vein graphite and characterization of the products. Sci. Rep..

[23] Zhao C.e., Wang W.J., Sun D., Wang X., Zhang J.R., Zhu J.J. (2014). Nanostructured Graphene/TiO_2_ Hybrids as High-Performance Anodes for Microbial Fuel Cells. Chem. Eur. J..

[24] Polverino S., Del Rio Castillo A.E., Brencich A., Marasco L., Bonaccorso F., Morbiducci R. (2022). Few-Layers Graphene-Based Cement Mortars: Production Process and Mechanical Properties. Sustainability.

[25] De Silva K.K.H., Huang H.H., Yoshimura M. (2018). Progress of reduction of graphene oxide by ascorbic acid. Appl. Surf. Sci..

[26] Stankovich S., Dikin D.A., Piner R.D., Kohlhaas K.A., Kleinhammes A., Jia Y., Wu Y., Nguyen S.T., Ruoff R.S. (2007). Synthesis of graphene-based nanosheets via chemical reduction of exfoliated graphite oxide. Carbon.

[27] Vacchi I.A., Ménard-Moyon C., Bianco A. (2017). Chemical Functionalization of Graphene Family Members. Phys. Sci. Rev..

[28] Wan X., Lu H., Kang J., Li S., Yue Y. (2018). Preparation of graphene-glass fiber-resin composites and its electromagnetic shielding performance. Compos. Interfaces.

[29] Tatarova E., Dias A., Henriques J., Abrashev M., Bundaleska N., Kovacevic E., Bundaleski N., Cvelbar U., Valcheva E., Arnaudov B. (2017). Towards large-scale in free-standing graphene and N-graphene sheets. Sci. Rep..

[30] Rao K.S., Senthilnathan J., Liu Y.F., Yoshimura M. (2014). Role of peroxide ions in formation of graphene nanosheets by electrochemical exfoliation of graphite. Sci. Rep..

[31] Cai T., Jiang N., Zhen G., Meng L., Song J., Chen G., Liu Y., Huang M. (2020). Simultaneous energy harvest and nitrogen removal using a supercapacitor microbial fuel cell. Environ. Pollut..

[32] Baudler A., Schmidt I., Langner M., Greiner A., Schröder U. (2015). Does it have to be carbon? Metal anodes in microbial fuel cells and related bioelectrochemical systems. Energy Environ. Sci..

[33] Feng H., Liang Y., Guo K., Chen W., Shen D., Huang L., Zhou Y., Wang M., Long Y. (2016). TiO_2_ Nanotube Arrays Modified Titanium: A Stable, Scalable, and Cost-Effective Bioanode for Microbial Fuel Cells. Environ. Sci. Technol. Lett..

[34] Chae K.J., Choi M., Ajayi F.F., Park W., Chang I.S., Kim I.S. (2008). Mass Transport through a Proton Exchange Membrane (Nafion) in Microbial Fuel Cells. Energy Fuels.

[35] Kalaiselvan N., Glivin G., Bakthavatsalam A., Mariappan V., Premalatha M., Raveendran P.S., Jayaraj S., Sekhar S.J. (2022). A waste to energy technology for Enrichment of biomethane generation: A review on operating parameters, types of biodigesters, solar assisted heating systems, socio economic benefits and challenges. Chemosphere.

[36] Khalid A., Arshad M., Anjum M., Mahmood T., Dawson L. (2011). The anaerobic digestion of solid organic waste. Waste Manag..

[37] Hassan A., Nelson B. (2012). Invited review: Anaerobic fermentation of dairy food wastewater. J. Dairy Sci..

[38] Ni G., Christel S., Roman P., Wong Z.L., Bijmans M.F., Dopson M. (2016). Electricity generation from an inorganic sulfur compound containing mining wastewater by acidophilic microorganisms. Res. Microbiol..

[39] Slonczewski J.L., Fujisawa M., Dopson M., Krulwich T.A. (2009). Cytoplasmic pH Measurement and Homeostasis in Bacteria and Archaea. Adv. Microb. Physiol..

[40] Logan B.E. (2007). Microbial Fuel Cells.

[41] Ji H., Sun H., Qu X. (2016). Antibacterial applications of graphene-based nanomaterials: Recent achievements and challenges. Adv. Drug Deliv. Rev..

[42] Sun M., Liu B., Yanagawa K., Ha N.T., Goel R., Terashima M., Yasui H. (2020). Effects of low pH conditions on decay of methanogenic biomass. Water Res..

[43] EG & G Technical Servoces, Inc (2004). Fuel Cell Handbook.

[44] Lew K. (2009). Acids and Bases.

[45] Ge Z., Zhang F., Grimaud J., Hurst J., He Z. (2013). Long-term investigation of microbial fuel cells treating primary sludge or digested sludge. Bioresour. Technol..

[46] Jang J.K., Pham T.H., Chang I.S., Kang K.H., Moon H., Cho K.S., Kim B.H. (2004). Construction and operation of a novel mediator- and membrane-less microbial fuel cell. Process Biochem..

[47] Jannelli N., Anna Nastro R., Cigolotti V., Minutillo M., Falcucci G. (2017). Low pH, high salinity: Too much for microbial fuel cells?. Appl. Energy.

[48] Dopson M., Ni G., Sleutels T.H. (2016). Possibilities for extremophilic microorganisms in microbial electrochemical systems. FEMS Microbiol. Rev..

[49] Borole A.P., O’Neill H., Tsouris C., Cesar S. (2008). A microbial fuel cell operating at low pH using the acidophile Acidiphilium cryptum. Biotechnol. Lett..

[50] Kim H., Kim B., Kim J., Lee T., Yu J. (2014). Electricity generation and microbial community in microbial fuel cell using low-pH distillery wastewater at different external resistances. J. Biotechnol..

